# MEDIATING EFFECT OF OCCUPATIONAL STRESS BETWEEN SKELETAL MUSCLE DISORDERS AND DEPRESSIVE SYMPTOMS IN FIREFIGHTERS: A STRUCTURAL EQUATION MODELING APPROACH

**DOI:** 10.13075/ijomeh.1896.02457

**Published:** 2025

**Authors:** Fengqiong Chen, Liang Wang, Jin Wang, Meiling Liu, Shiwei Cao, Yunxuan Huang, Huaxin Deng, Mengliang Ye

**Affiliations:** 1 Chongqing Center for Disease Control and Prevention, Chongqing, China; 2 Chongqing Medical University, College of Public Health, Chongqing, China; 3 Chinese Center for Disease Control and Prevention, Institute of Occupational Health and Poisoning Control, Beijing, China; 4 The First Affiliated Hospital of Chongqing Medical University, Department of Gastroenterology, Chongqing, China; 5 Chongqing Medical University, The Second Clinical College, Chongqing, China

**Keywords:** depressive symptoms, occupational stress, firefighters, structural equation modeling, mediating effect, skeletal muscle disorders

## Abstract

**Objectives:**

The purpose of this study was to explore the relationship between skeletal muscle disorders (SMD), occupational stress (OS) and depressive symptoms (DS) among firefighters.

**Material and Methods:**

A cross-sectional survey was conducted among firefighters in Chongqing, China. Descriptive statistic and correlation analyses were performed by using SPSS 26.0. AMOS 24.0 was used to construct the structural equation modeling between SMD and DS. The mediating effect of OS was also evaluated.

**Results:**

The results demonstrate that SMD can predict DS (β = 0.25, p < 0.001) and OS (β = 0.39, p < 0.001) positively, OS positively predicted DS (β = 0.39, p < 0.001) after controlling for SMD. Additionally, OS has a partial indirect effect between DS and SMD (indirect effects = 0.209, 95% CI: 0.093–0.372, p < 0.001).

**Conclusions:**

Occupational stress has a partial indirect effect between SMD and DS among firefighters. Skeletal muscle disorders could affect DS not only directly, but also indirectly, by affecting OS. These findings may be of great significance and contribution to the future research of firefighters’ occupational health.

## INTRODUCTION

The World Health Organization claimed that depression might become the second major factor of disability around the world by 2020 [1]. About 6.9% of the Chinese population suffer from depression [2]. Depressive symptoms (DS) include mood disorders characterized by reduced sleep and appetite, feelings of disappointment, lack of interest, persistent low mood, and feelings of worthlessness [3]. Firefighters, as a specific body of emergency services personnel, must not only confront hazardous conditions but also undertake extensive training. Research has concluded that in China [4], at least 4% of firefighters experience psychological difficulties, such as loss of interest, anxiety, depression etc. and the incidence of depression in firefighters reached 22% [5]. Studies indicate that the primary source of physical and mental stress in firefighters might be their profession [6,7] and report an increased risk of physical and mental health problems in this group [8]. A large number of studies focus on investigating depression among doctors, nurses, teachers, and other high-stress occupations during the COVID-19 pandemic. However, limited consideration is given to the issue of DS in the high-risk occupation of firefighters.

Skeletal muscle disorders (SMD) refer to minor injuries that occur and accumulate in muscles, joints, blood vessels, or nerves during work due to long-term, repetitive movements and poor posture [9]. Physiological and psychological factors determine the development of SMD, which arise in firefighters of all age groups and become more pronounced with age [10]. Studies have shown that as SMD become more severe, they can induce DS and lead to poor physical health among workers [11]. The most common on-duty injuries are musculoskeletal injuries, such as muscle pain, accounting for between 43% and 62% of all injuries, which can further result in occupational stress (OS) and DS [12,13]. However, few studies have addressed the association between SMD and DS among firefighters, and the authors speculate that this connection is present.

The definition of OS is the physiological and psychological reaction that appears when the demands of a person's job position are not aligned with their ability and resources [14]. Occupational stress has become one of the most common work-related health concerns worldwide [15]. Previous studies have demonstrated that chronic exposure to excessive pressure at work may result in physical and mental damage [16], e.g., it can trigger fatigue [17], cardiovascular diseases, digestive diseases, immune system diseases, musculoskeletal disorders and anxiety symptoms [18]. Skeletal muscle disorders involve pain in the neck, back, shoulders, arms, etc., in particular, a potential cause of OS, which can lead to DS [19]. Occupational stress has a stronger impact on DS in occupational populations [20], and a decrease in OS could be effective in preventing DS in workers [21]. One study has demonstrated that OS rate among firefighters is significantly higher than in other professions [22].

According to available data, the innovation in this study is to explore the impact of SMD on DS, especially with OS as a mediating effect can fill the gap in previous studies, and the group of firefighters is relatively new. Chronic SMD can cause OS, while excessive OS can lead to psychological disorders, leading to DS. However, the relationship between these 3 factors among firefighters is still unclear, especially the mediating effect of OS between SMD and DS, so it is necessary to explore the impact of SMD on DS in Chinese firefighters through OS.

The mediating role of OS between SMD and DS has not been proven, but significant correlation between these factors has also been found [23]. Structural equation modeling (SEM) provides a comprehensive toolbox to analyze the multivariate interrelationships between directly observed variables and underlying structures [24], which is widely applied to complex phenomena in various areas such as the environment, health and humanities. It exhibits a multitude of advantages and benefits: firstly, measurement errors can be controlled. Secondly, mediation variables can be drawn upon easily. Thirdly, it can be used to statistically evaluate theoretical models. In this research, the authors will use SEM to verify the mediation effect of OS on SMD and DS, which could bridge the gap between previous studies.

The authors propose the following 3 hypotheses (H) based on the analysis presented above:

– H1: Firefighters who suffer from higher SMD are more likely to suffer from OS.
– H2: Firefighters who suffer from higher OS are more likely to suffer from DS.
– H3: Among firefighters, OS plays a mediating role between SMD and DS.

## MATERIAL AND METHODS

### Participants and processing procedure

A cross-sectional study of firefighters in Chongqing was conducted in 2020, and quantitative investigation is supplemented by qualitative investigation. The authors adopted a simple random sampling method to choose firefighters for this study, and distributed questionnaires to them through the WeChat platform. In order to enhance sample coverage and reduce potential bias, the authors picked firefighters from varied districts of Chongqing, which enhances the representativeness of this research in Chongqing. A total of 450 questionnaires had been sent and 423 were submitted, 16 of which had missing values or abnormal data, while the remaining 407 were valid. In regression analysis, the required sample size is usually >10 times the number of independent variables [25]. The number of participants should be at least 70 as much as this study involved 7 independent variables, and the authors ultimately obtained 407 valid questionnaires, which was sufficient for statistical analysis. The participants also fulfilled the following selection criteria:

– no history of mental illness, and no use of psychotropic drugs,
– having worked in their current role for >6 months
– participating in the survey voluntarily and having signed the informed consent form.

Exclusion criteria:

– recent use of psychotropic drugs,
– resignation or long-term sick leave
– having worked in their current role for <6 months,
– refusal to participate in the investigation.

The project was approved by the Medical Ethical Review Committee, National Institute for Occupational Health And Poison Control, Chinese Center for Disease Control and Prevention (grant No. NIOHP201914).

The study participants were 407 firefighters from Chongqing whose age was mean (M) ± standard deviation (SD) 29.12±5.86 years, of which 392 (96.3%) were male and 15 (3.7%) were female. Their scores for OS and SMD were M±SD 44.9±9.8 (male), 47.7±5.9 (female) and M±SD 3.1±2.9 (male), 1.9±2.1 (female), respectively. Furthermore, their detection rates of DS were 22.7% and 40%, respectively. The prevalence of DS was reported as 26.1% in males and 28.7% in females in Korea [26]. Their education level was mostly vocational college or junior college, which accounted for 44.2%. The majority of individuals, 67.1%, were unmarried. Furthermore, most firefighters were young, with 22.4% of firefighters being <25 years of age and 65.1% falling within the age range of 25–35 years.

### Measurement

#### Basic demographic information

Individuals participating in this study were required to provide complete and accurate information, including on their gender, educational level, marital status, and age. Gender is used as a control variable in this study. Table 1 presents specific information on these variables.

**Table 1.** Comparison of occupational stress, depressive symptoms and skeletal muscle disorders under different demographic characteristics in firefighters, Chongqing, China, 2020

Variable	Participants (N = 407)		Depressive symptoms			Occupational stress		Skeletal muscle disorders	
	n	%	yes [n (%)]	no [n (%)]	χ2	score (M±SD)	t/F	score (M±SD)	t/F
Gender					2.415		1.196		2.271
male	392	96.3	89 (22.7)	303 (77.3)		44.9±9.8		3.1 ±2.9	
female	15	3.7	6 (40.0)	9 (60.0)		47.7±5.9		1.9±2.1	
Education level					4.149		1.167		0.685
junior high school and below	12	3.0	2 (16.7)	10 (83.3)		43.8±7.2		2.3±3.4	
high school or technical secondary school	158	38.8	32 (20.3)	126 (79.3)		43.6±9.9		2.9±3.0	
junior college or vocational college	180	44.2	43 (23.9)	137 (76.1)		46.1 ±9.5		3.2±3.0	
undergraduate	53	13.0	16 (30.2)	37 (69.8)		45.4±10.3		3.3±2.4	
postgraduate or above	4	1.0	2 (50.0)	2 (50.0)		46.8±6.3		2.3±2.1	
Marital status					8.014*		3.775*		0.654
unmarried	273	67.1	56 (20.5)	217 (79.5)		44.2±9.9		3.0±3.0	
married	115	28.3	31 (27.0)	84 (73.0)		46.0±8.6		3.0±2.8	
divorced/widowed	19	4.6	8 (42.1)	11 (57.9)		49.7±11.3		3.8±2.6	
Age					1.869		5.401**		1.617
≤25 years	91	22.4	17 (18.7)	74 (81.3)		41.7±10.5		2.8±3.2	
26–35 years	265	65.1	64 (24.2)	201 (75.8)		46.1 ±9.7		3.2±2.9	
36–45 years	38	9.3	11 (28.9)	27 (71.1)		46.0±7.0		3.0±3.0	
≥46 years	13	3.2	3 (23.1)	10 (76.9)		42.0±6.9		1.6±1.7	
Total			95 (23.3)	312 (76.7)		45.0±9.7		3.1 ±2.9	

F – F-test; t – Students’ t-test.

** p < 0.01;

* p < 0.05.

#### Assessment of occupational stress

The *Core Occupational Stress Scale* (COSS) was designed by the project team to calculate the OS of the participants, which is a new OS evaluation method for Chinese occupational populations established by the National Institute of Occupational Health and Poisoning Control [27]. This scale consisted of 17 items, which consisted of 4 OS related dimensions: organizational reward, social support, autonomy, demand and effort. Every item used the 5-point Likert scale: “strongly agree” (5 pts), “agree”(4 pts), “basically agree” (3 pts), “disagree” (2 pts) and “completely disagree” (1 pt). The items related to “social support” and “autonomy” were scored in reverse (6 − actual score), and the total score was calculated by adding the scores of each dimension. The total score was directly proportional to the severity of OS situation, with the higher total scores indicating more severe OS. This Cron-bach's α of the scale was 0.808.

#### Assessment of depressive symptoms

The assessment of DS was performed using the translated *Patient Health Questionnaire* (PHQ-9), containing 9 items, which has been verified as credible and effective in the Chinese population [28]. It evaluated the frequency of symptoms over the past 2 weeks and each item was scored on a 4-point Likert scale: “all the time” (3 pts), “more than a half” (2 pts), “no more than a half” (1 pt), “never” (0 pt), and when total score exceeded 10 pts, the respondent was considered to have DS. Moreover, the DS was consider more severe with higher scores, and the Cronbach's α of this scale was 0.927 in this study.

#### Assessment of skeletal muscle disorders

An integrated scale based on the *Dutch Musculoskeletal Questionnaire* and the *Nordic Standard Questionnaire of Work-related Musculoskeletal Disorders* [29] after translation and modification by Lei Yang et al. [30–32] was used to evaluate work-related SMD. This questionnaire has been extensively used in SMD research for the different occupational groups and the general population. The scale measures 9 parts of the body: shoulder, elbow, neck, waist, back, knee, wrist, ankle, and hip. One mark is given for a “yes” answer, 0 marks for a “no” answer. Higher scores indicate more severe symptoms. This Cronbach's α of this scale is 0.905. Table 2 presents the validity and reliability condition of all scales.

**Table 2.** Reliability and validity test of occupational stress, depressive symptoms and skeletal muscle disorders scales in firefighters, Chongqing, China, 2020

Variable	Cronbach's α	KMO	p	Items [n]
Occupational stress	0.808	0.861	<0.01	17
Depressive symptoms	0.927	0.936	<0.01	9
Skeletal muscle disorders	0.876	0.889	<0.01	9

KMO – the Kaiser Meyer Olkin test.

### Data processing and statistical analysis

After removing invalid records with missing data, duplicates, and blank items, 407 complete data sets were included in the final analysis. The authors used SPSS 26.0 for data analysis and normality testing, M±SD were used to represent continuous variables that follow a normal distribution, and the error distribution was also normal. Categorical variables are represented in terms of frequency and percentage. Then the authors conducted a one-way analysis of variance (ANOVA), and Pearson correlation analysis. The χ^2^ test and independent sample t-test were used to test the difference in scores under different covariates. Structural equation modeling was constructed by AMOS 24.0, and a mediation effects analysis was also performed. P < 0.05 indicates a statistically significant result in this study.

## RESULTS

### Chi-square test and t-test

There are significant differences between DS and OS in relation to marital status (p < 0.05), OS exhibits a clear difference across ages (p < 0.01). More detailed information is demonstrated in Table 1.

### Correlation analysis

The Pearson's correlation analysis demonstrated that the OS of firefighters correlated with DS positively (r = 0.443, p < 0.01), and SMD also associated with DS positively (r = 0.357, p < 0.01). Besides, OS had a positive association with SMD (r = 0.337, p < 0.01). Table 3 presents the correlation of other unambiguous dimensions.

**Table 3.** Pearson correlation analysis across occupational stress, depressive symptoms and skeletal muscle disorders in firefighters, Chongqing, China, 2020

Variable	Pearson's correlation											
	1	2	3	4	5	6	7	8	9	10	11	12
1. Occupational stress scores	1											
2. Skeletal muscle disorders	0.337**	1										
3. Interests	0.367**	0.246**	1									
4. Mood	0.337**	0.291**	0.646**	1								
5. Sleep	0.388**	0.287**	0.575**	0.651**	1							
6. Fatigued	0.410**	0.376**	0.652**	0.722**	0.743**	1						
7. Appetite	0.376**	0.295**	0.593**	0.628**	0.663**	0.725**	1					
8. Failure	0.269**	0.239**	0.560**	0.662**	0.570**	0.631**	0.596**	1				
9. Attention	0.364**	0.308**	0.559**	0.619**	0.572**	0.631**	0.563**	0.615**	1			
10. Action	0.362**	0.330**	0.579**	0.602**	0.576**	0.619**	0.593**	0.623**	0.700**	1		
11. Suicide	0.267**	0.145**	0.427**	0.432**	0.376**	0.335**	0.414**	0.487**	0.422**	0.445**	1	
12. Depressive symptoms	0.443**	0.357**	0.785**	0.835**	0.814**	0.860**	0.818**	0.805**	0.803**	0.808**	0.581**	1

** p < 0.01.

### Descriptions of assumed model and mediating effect

**Figure 1.**
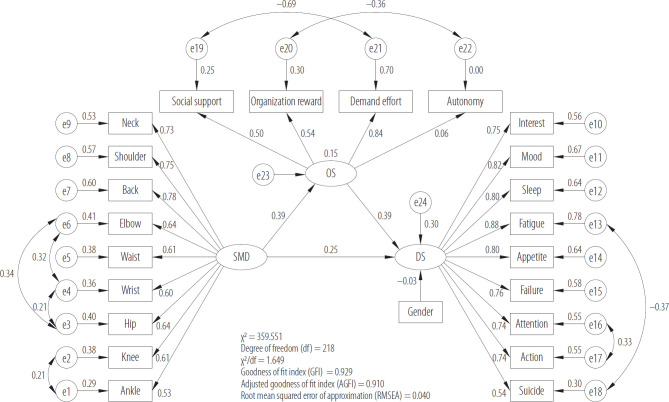
Structural equation modeling of occupational stress (OS), depressive symptoms (DS) and skeletal muscle disorders (SMD) in firefighters, Chongqing, China, 2020

In the light of the correlation analysis, SEM was employed to explore the relationship between OS, SMD and DS. Skeletal muscle disorders were regarded as the independent variable (X), OS was the mediator variable (MED), and the DS was the dependent variable (Y) in the model (Figure 1).

e1~e24 is residual error.

The fitting index of the hypothetical model is shown in Table 4. The χ^2^ minimum (CMIN)/degree of freedom (df) is 1.649 (it is better when the value is <3), root mean squared error of approximation (RMSEA) is 0.040 (in general, the required value is <0.08, this is <0.05, values <0.05 are deemed as a “close fit” [33], indicating a good fit of the model), standardized root mean square residual (SRMR) = 0.0398 (when it is <0.05 it is considered that the model fitting effect is good), goodness of fit index (GFI) is 0.929, adjusted goodness of fit index (AGFI) is 0.910, Tucker-Lewis index (TLI) is 0.961, normative fit index (NFI) is 0.920, incremental fit index (IFI) is 0.967, comparative fit index (CFI) is 0.967, parsimony fit index (PGFI) is 0.734, adjusted normative fit index (PNFI) is 0.793 (value >0.50 indicates better performance). The criterion of TLI, NFI, AGFI, GFI, IFI, and CFI is >0.9 [34]. Each parameter of the model fitting meets the standard and is significant, so it can be considered that the model fitting effect is better.

**Table 4.** Model fit index of structural equation modeling in firefighters, Chongqing, China, 2020

Index	Reference standard	Value
CMIN/df	1–3 is excellence	1.649
RMSEA	<0.05 is excellence	0.040
SRMR	<0.05 is good	0.040
GFI	>0.9 is good	0.929
AGFI	>0.9 is good	0.910
TLI	>0.9 is good	0.961
IFI	>0.9 is good	0.967
NFI	>0.9 is good	0.920
CFI	>0.9 is good	0.967
PGFI	>0.5 is good	0.734
PNFI	>0.5 is good	0.793

AGFI − adjusted goodness of fit index; CFI − comparative fit index; adjusted normative fit index; CMIN/df − χ^2^ fit statistics/degree of freedom; GFI − goodness of fit index; IFI − incremental fit index; NFI − normative fit index; PGFI − parsimony goodness of fit index; PNFI − parsimony normed fit index; RMSEA − root mean squared error of approximation; SRMR − standardized root mean square residual;TLI −Tucker-Lewis index.

The results for the mediating effect demonstrate that SMD can affect DS (β = 0.25, p < 0.01) and OS (β = 0.39, p < 0.01) positively, OS affected DS positively (β = 0.39, p < 0.01) after controlling for SMD. The confidence interval (CI) of the mediating effect is 0.093–0.372, the mediating effect is deemed significant when the CI does not include 0 [35]. Therefore, the mediating effect of the theoretical model is significant. The indirect effects are 0.209 (95% CI: 0.093–0.372, p < 0.001), direct effects are 0.697 (95% CI: 0.435–1.013, p < 0.001), and the total effects are 0.906 (95% CI: 0.651–1.238, p < 0.001). Table 5 shows the specific values of the whole effect. Furthermore, the equation of the mediating effect model is as follows:



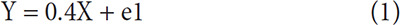





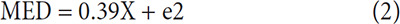





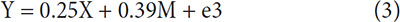



where:

X – the independent variable,

MED – the mediator variable,

Y − the dependent variable,

e1, e2, e3 − random error of 3 equations.

**Table 5.** Mediating effects of skeletal muscle disorders and depressive symptoms in firefighters, Chongqing, China, 2020

Effects	Value	SE	p	β	95% CI
Total	0.906	0.149	<0.001	0.4	0.651–1.238
Direct	0.697	0.145	<0.001	0.25	0.435–1.013
Indirect	0.209	0.072	<0.001	0.39	0.093–0.372

## DISCUSSION

As is widely known, firefighters are often required to respond to emergency situations, which inevitably brings tremendous psychological pressure, thereby exposing them to higher risks of mental disorders [36]. Moreover, the occurrence of SMD resulting from training programs can further contribute to objective stress scores. This phenomenon is consistent with the findings of this study, the significance of which lies in the area of preventing the occurrence of OS and DS among firefighters through a new perspective, which involves a reduction in SMD incidence. Firefighters often experience body soreness after engaging in prolonged, repetitive, and rigorous training, SMD may be associated with repetition and demanding work [37]. A significant percentage of firefighters − 40%, report having musculoskeletal issues, and back, shoulder, and knee problems are the most typical symptoms [38]. In addition, the authors found that married people suffer from lower DS levels than those who are not married, because they are more likely to receive support from their families and spouses, which reduces DS incidence. However, in addition to work pressure, they constantly worry about supporting their families, which may result in higher OS ratings than for single people. Accordingly, previous studies have demonstrated that OS is also a decisive factor in marital satisfaction [39]. This means that intervention can be made in the area of family support and understanding to reduce the incidence of OS among firefighters and thus diminish the occurrence of DS. Moreover, age also affects OS in different ways, with those <25 years of age having lower OS ratings, which are often correlated with reduced family and marital obligations. Due to the stress of starting a family and beginning employment, people aged 25–35 years had the highest OS ratings. After the age of 35 years, life becomes more stabilized, with fewer fluctuations, so the OS scores begin to decrease significantly.

The research results indicated that SMD correlated positively with DS and OS. Previous studies have implied that firefighters’ SMD were one of the reasons for OS [40], and SMD pain can make it uncomfortable for firefighters to work, which causes OS. Also, numerous studies consider OS a risk factor for developing DS [41,42]. The authors’ research findings not only confirm the relationship between SMD and OS, as former studies have shown, but also propose a new perspective on the potential mechanism of OS between firefighters’ SMD and DS. The more severe the OS becomes, the higher the levels of anxiety and distress may be experienced by firefighters, making them more likely to suffer from DS. This corroborates hypotheses H1 and H2.

The authors also explored how SMD not only affect DS directly, but also act on DS by affecting OS indirectly. The mediation test results of SEM are significant, OS plays a partial mediating role between SMD and DS. This means that firefighters who experience increased SMD are more likely to suffer from higher levels of OS, which adds to the risk of developing DS [43]. Therefore, H3 is also true.

Generally, firefighters remain in a constant state of preparedness for dangerous situations and uncertain events over extended periods of time [44], so they may be worried and afraid of serious incidents [45]. In addition, firefighters are required to work shifts and receive less support, which can cause OS and affect emotions, leading to the emergence of DS. As a result, it is crucial for firefighters to assess and manage their negative emotions properly. Most firefighters have an average level of mental health and some of them are in a distressed mental state [46]. This phenomenon was particularly prevalent among young firefighters. In the authors’ study, >23% of firefighters experienced DS, a slightly higher value than in other studies, which suggests that in real life DS may be much more severe than it may be perceived.

These results indicate there is a significant correlation among the 3 factors in question, and the mediating effect of OS between SMD and DS was verified. The findings present an opportunity to increase care and attention given to firefighters. By rationally arranging their work schedules and providing them with regular psychological counseling, it is possible to alleviate their OS and DS. As the authors revealed the incidence of these occupational health problems among firefighters, action must be taken to counteract them. First of all, local governments should conduct regular physical examinations and psychological counseling for firefighters. Secondly, the authorities should consider promoting the visibility of fire protection work to increase its public awareness, so as to better support the work of firefighters. Finally, it is also essential for firefighters themselves to perform strength training exercise in their spare time and increase efforts to improve their psychological constitution and stress resistance. By doing this, they can achieve good physical health, but also better mental health.

## CONCLUSIONS

The authors’ research hypotheses have been confirmed and there is a close correlation between the SMD, OS, and DS of firefighters. In particular:

– the probability of OS among firefighters increases with more severe SMD,
– the probability of DS in an individual increases with high OS scores,
– OS plays a mediating effect between SMD and DS, which has not been examined in previous studies.

This discovery provides a new perspective on the prevention of and intervention in occupational health problems and mental health issues among firefighters.

### Limitations

Although all the hypotheses have been confirmed, several limitations of this study must be addressed in the future. First and foremost, in terms of collection, the results of online questionnaires may be arbitrary, contributing to a certain bias in data collection. Secondly, in the aspects of sample and data analysis, the sample size for this study may not be large enough, so it should be expanded and the diversity of the studied population should be increased in subsequent studies.
